# Characterization and Antimicrobial Activity of the Teleost Chemokine CXCL20b

**DOI:** 10.3390/antibiotics9020078

**Published:** 2020-02-12

**Authors:** Xun Xiao, Yanqi Zhang, Zhiwei Liao, Jianguo Su

**Affiliations:** 1 Department of Aquatic Animal Medicine, College of Fisheries, Huazhong Agricultural University, No. 1 Shizishan St., Wuhan 430070, China; xiaoxunhn@163.com (X.X.); yanqizhang2016@163.com (Y.Z.); liaozhiwei1991@163.com (Z.L.); 2 Laboratory for Marine Biology and Biotechnology, Pilot National Laboratory for Marine Science and Technology, Qingdao 266237, China

**Keywords:** chemokine, *Ctenopharyngodon idella*, antimicrobial peptide, membrane permeabilization

## Abstract

Fish are a potential source of diverse organic compounds with a broad spectrum of biological activities. Many fish-derived antimicrobial peptides and proteins are key components of the fish innate immune system. They are also potential candidates for development of new antimicrobial agents. CXCL20b is a grass carp (*Ctenopharyngodon idella*) CXC chemokine strongly transcribed at the early stage of bacterial infections, for which the immune role had not been reported to date. In the present study, we found that CXCL20b is a cationic amphipathic protein that displays potent antimicrobial activity against both Gram-positive and Gram-negative bacteria. The results of DiOC_2_(3) and atomic force microscopy (AFM) assays indicated that CXCL20b could induce bacterial membrane depolarization and disruption in a short time. By performing further structure-activity studies, we found that the antimicrobial activity of CXCL20b was mainly relative to the N-terminal random coil region. The central part of this cytokine representing β-sheet region was insoluble in water and the C-terminal α-helical region did not show an antimicrobial effect. The results presented in this article support the poorly understood function of CXCL20b, which fulfills an important role in bony fish antimicrobial immunity.

## 1. Introduction

The innate immune system, largely based on antimicrobial peptides (AMPs) [1], provides a front line of defense against invading bacterial pathogen. There are over one hundred human AMPs have been reported [2]. The major members include cathelicidins [3], defensins [4], histatins [5], RNases [6], dermcidin [7], and antimicrobial chemokines [8]. In general, AMPsare characterized by a net positive charge (cationic) and segregated regions of polar and nonpolar residues (amphipathicity) [9]. AMPscan selectively permeate bacterial membranes and kill via disruption of the barrier function. The increasing incidence of infections resulting from multiple drug-resistant pathogens in clinical settings has intensified the demand for alternative therapies. AMPs with potent antimicrobial activities and diverse mechanisms of action are considered important alternatives to solving the issues of multiple drug-resistance [10].

The chemokine super family is important for regulating the migration of various immune cells [1,11]. Mammals have approximately 50 chemokine ligands, and most of them are 5-10 kDa small proteins, which have four cysteine residues [12]. On the basis of the distinctive pattern of the first two cysteine residues, four subfamilies are categorized as CXC, CC, C, and CX3C classes [13]. In addition to their cytokine signaling activity, parts of mammalian chemokines are also potent broad-spectrum antimicrobial proteins called kinocidins [14]. These antimicrobial chemokines are important in host defense against bacterial pathogens.

Besides being considered a food source, non-venomous and non-poisonous fish could also be considered as a potential source of a variety of biologically active substances, including polyunsaturated fatty acids, carbohydrates, enzymes, and peptides [15]. In previous studies, many bony fish chemokine genes were identified [16]. To date, four subfamilies, namely, CXC, CC, CX, and C, have been identified among fish chemokines [13]. The CXC subfamily is one of the largest subfamilies in both mammals and fish. In mammals, the CXC subfamily consists of 17 chemokines [17], but each species has different numbers of genes: 16 members for humans (lacking CXCL15), 15 members for mice (lacking CXCL8 and CXCL6), a small number for other mammalian species, 17 CXC-type members for catfish (*Ictalurus punctatus*) [13], 23 members for zebrafish (*Danio rerio*) [18], and 20 members for grass carp (*Ctenopharyngodon idella*) [19]. Extensive phylogenetic analysis established nine major groups of highly related CXC chemokines in bony fish [20]; these groups are CXCL8, CXCL11, CXCL12, CXCL13, CXCL14, CXCL18, CXCL19, CXCL20, and CXCL32 [21]. In particular, CXCL18, CXCL19, CXCL20, and CXCL32 are fish-special groups that have low homology to mammalian chemokines [22].

To date, the function of the CXCL11 [23], CXCL8 [24], and CXCL12 [25] groups has been widely studied in bony fish. Recently, many studies have indicated that bony fish CXCL20 group (especially CXCL20b) may play an important role in host defense against bacterial infections. For instance, the mRNA expression levels of catfish CXCL20a and CXCL20b were remarkably higher in resistant fish than that in susceptible fish after *Edwardsiella ictaluri* and *Flavobacterium columnare* infections [18]. In addition, our previous study demonstrated that grass carp CXCL20b is one of the few significantly upregulated chemokines during the early stage of *Aeromonas hydrophila* infection [19]. However, in the context of bacterial infections, the function of grass carp CXCL20b remains poorly understood. 

In the present study, we produced recombinant grass carp CXCL20b. To our surprise, 3D modeling and net charge analysis showed that grass carp CXCL20b is an unusual cationic and amphipathic protein. These untypical structure features of grass carp CXCL20b urged us to further investigate its potential antimicrobial function, antimicrobial mechanism, and possible antimicrobial active region. In this article, we showed that CXCL20b could efficiently kill bacteria by causing bacterial membrane depolarization and disruption. Furthermore, our data indicated that the antimicrobial activity of CXCL20b might be largely related to its N-terminal region peptides. Overall, our study strongly supported that grass carp CXCL20b might play a pivotal role in antimicrobial immunity by serving as a direct antimicrobial protein.

## 2. Results

### 2.1. Producing of Recombinant Grass Carp CXCL20b

**Figure 1:**
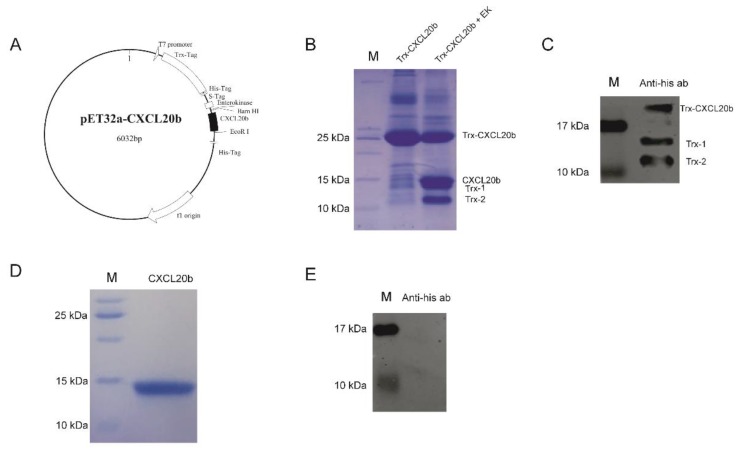
Producing of recombinant grass carp CXCL20b. (**A**) Plasmid map of recombinant pET32a-CXCL20b. pET32a-CXCL20b consisted of some small tags (3 kDa), large Trx tag (16.7 kDa), and CXCL20b sequence (7.6 kDa). (**B**) SDS-PAGE analysis of the Ni-affinity chromatography purification and enzyme digestion of Trx-CXCL20b. Lane M: protein molecular weight mark. Lane Trx-CXCL20b: purified Trx-CXCL20b protein from the Ni-NTA column. Lane Trx-CXCL20b + EK: enterokinase digestion of purified Trx-CXCL20b protein. (**C**) Western Blot (WB) analysis of the cleavage mixture with Anti-6 × His antibody. Lane M: protein molecular weight mark. Lane Anti-his ab: enterokinase digestion of purified Trx-CXCL20b protein. (**D**) SDS-PAGE analysis of the purified CXCL20b protein. Lane M: protein molecular weight mark. Lane CXCL20b: purified CXCL20b protein from the Hitrap SP FF column. (**E**) WB analysis of the purified CXCL20b protein with Anti-6 × His antibody. Lane M: protein molecular weight mark. Lane Anti-his ab: purified CXCL20b protein from the Hitrap SP FF column.

To investigate the function of grass carp CXCL20b, we produced the recombinant grass carp CXCL20b protein firstly. To our knowledge, most functional mammalian chemokines can be produced through an *Escherichia coli* expression system [26]. To avoid the difficult renaturation process of inclusion bodies, a practical method to obtain a soluble protein is the fusion of the target protein with a tag, which can facilitate purification without affecting the properties of the target protein [27]. Here, by using prokaryotic expression vector pET32a, grass carp CXCL20b (approximately 7.6 kDa) was fused with a 6 × His tag (approximately 1.1 kDa) and Trx tag (approximately 16.7 kDa) at its N-terminal (Figure 1A). By Ni-affinity chromatography, we obtained soluble Trx-CXCL20b (approximately 25 kDa) protein (the purity of Trx-CXCL20b >70% by SDS-PAGE analysis; Figure 1B and Supplemental Figure S1A). The purified fusion proteins were then treated with enterokinase to obtain cleaved CXCL20b. Although no cleavage site for enterokinase is present in the Trx protein, some additional bands possibly due to non-specific cleavage of the fusion protein were observed (Figure 1B). In particular, the apparent molecular weight of cleaved CXCL20b was about 14 kDa, which was bigger than the theoretical molecular weight (Figure 1B). It is unexpected, hence we used the Anti-6 × His Tag mouse monoclonal antibody to label the cleavage mixture by Western Blot (WB). Consistently, the additional 14 kDa band detected by Coomassie blue staining is the only protein bind, which could not be detected by Anti-6 × His Tag mouse monoclonal antibody (Figure 1C). This result indicated that the Trx-1 and Trx-2 are the products of enterokinase after non-specific restriction of Trx tag protein. The cleaved CXCL20b could readily be further purified from the cleavage mixture by cation exchange chromatography. In this case, we collected a single CXCL20b elution peak in the eluent of 150–200 mM sodium chloride concentration (unpublished data). The purified grass carp CXCL20b was detected by Coomassie blue staining (Figure 1D and Supplemental Figure S1B) and WB (Figure 1E), and the protein purity was over 90%.

### 2.2. 3D Modeling and Net Charges Distribution Analysis of CXCL20b

**Figure 2:**
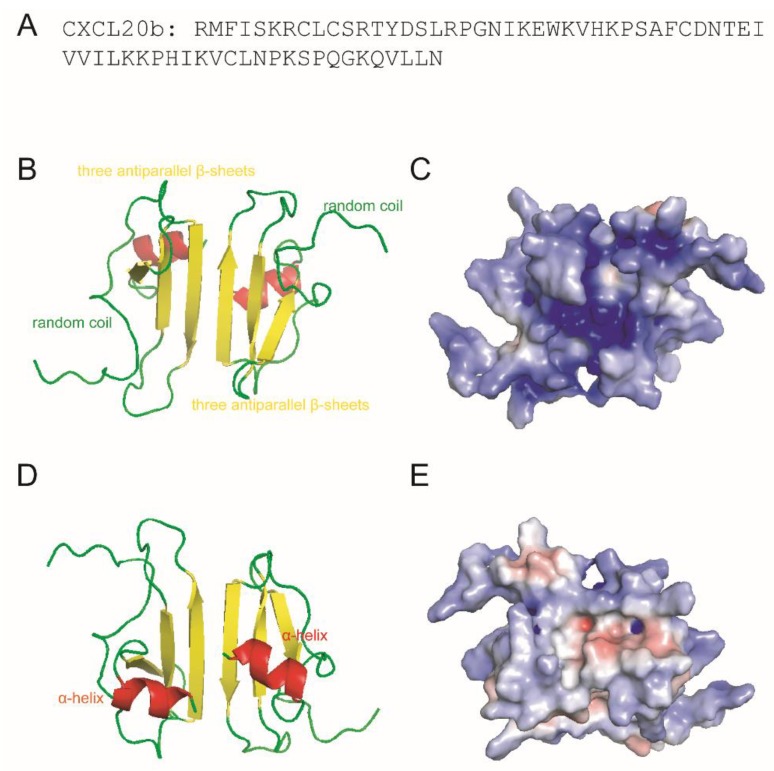
3D modeling and surface charge distribution analysis of grass carp CXCL20b. (**A**) The amino acids sequence of CXCL20b. (**B**,**D**) On the basis of the prediction of grass carp CXCL20b, the images show a 3D model of CXCL20b dimers. Grass carp CXCL20b features a typical mammalian chemokine structure, including N-terminal random coil (green), core structure containing three antiparallel β-strands (**yellow**), and C-terminal α-helix (**red**). (**C**,**E**) Electrostatic potentials mapped onto the surfaces of grass carp CXCL20b. Blue represents the cationic regions, red represents the negative regions, and white represents the hydrophobic residues.

Our sequence analysis (Figure 2A) of grass carp CXCL20b indicated the potent cationic nature of this protein (the calculated charge of CXCL20b was +9.0 at pH 7 and isoelectric point of 10.4) [19]. 3D modeling analysis of grass carp CXCL20b showed that this cytokine featured a typical mammalian chemokine structure, including N-terminal random coil, core structure containing three antiparallel β-strands, and C-terminal α-helix (Figure 2B). In particular, the structure prediction showed that the homology model of grass carp CXCL20b had a 3D structure very similar to the NMR structure of human chemokine CXCL1 dimer [28] (Supplemental Figure S2A). Subsequently, we analyzed the distribution of cationic amino acid residues across the protein surface. We found that most cationic charges were focused on the surface of CXCL20b dimer, which contained the most antiparallel β-strands and N-terminal random coil region (Figure 2C). On the opposite side, two hydrophobic protrusions that were mainly composed of a C-terminal α-helix region were found (Figure 2D). By contrast, CXCL20b presented fewer positive charges and irregular distribution across this molecular surface (Figure 2E). Overall, the hydrophobic amino acids on one side of the CXCL20b dimer and cationic residues on the opposite side suggested that grass carp CXCL20b dimer was a cationic amphipathic molecule. Interestingly, the amphipathic and cationic properties were also typical characteristics of human chemokine CXCL1 (Supplemental Figure S2B,C). Since human CXCL1 had been proved to be a potent antimicrobial chemokine in innate immunity [14], we asked whether grass carp CXCL20b has similar functions.

### 2.3. Antimicrobial Activity Assay of CXCL20b

**Figure 3:**
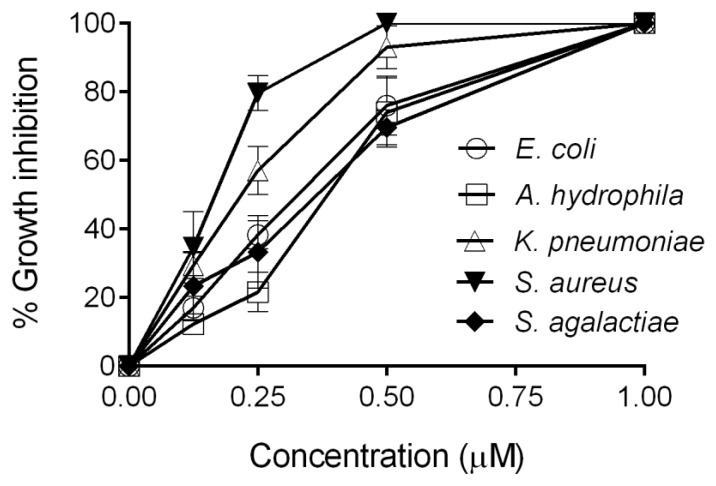
Antimicrobial activity of grass carp CXCL20b. The antimicrobial activity of grass carp CXCL20b was determined through CFU assays. Increasing concentrations of recombinant grass carp CXCL20b protein were incubated for 30 min with Gram-positive bacteria *Staphylococcus aureus* (ATCC 25923), *S. agalactiae* (ATCC 13813), Gram-negative bacteria *Escherichia coli* (ATCC 25922), *Aeromonas hydrophila* (ATCC 7966), and *Klebsiella pneumoniae* (K13). Data from three independent experiments are shown (mean ± SEM).

Given that amphipathic and cationic nature are typical characteristics of many AMPs and kinocidins [29], we asked whether grass carp CXCL20b has direct antimicrobial function. As shown in Figure 3, we tested the antimicrobial activity of recombinant grass carp CXCL20b protein against five representative bacterial strains in 30 min. We found that grass carp CXCL20b exerted direct antimicrobial activity against all the tested bacteria, including two Gram-positive bacteria *Staphylococcus aureus* (ATCC 25923) and *S. agalactiae* (ATCC 13813) and three Gram-negative bacteria *E. coli* (ATCC 25922), *A. hydrophila* (ATCC 7966), and *K. pneunoniae* (K13), under 30 min of treatment (Figure 3). Among these tested strains, recombinant grass carp CXCL20b protein showed the most efficient antimicrobial activity against *S. aureus* (Figure 3). The MBC_90_ for grass carp CXCL20b protein was also determined, as shown in Table 1. After 1 h of treatment with 0.25–0.5 µM CXCL20b protein, more than 90% of the tested bacteria were killed. These data indicated that grass carp CXCL20b is a potent antimicrobial chemokine.

### 2.4. DiOC_2_(3) Assay

**Figure 4:**
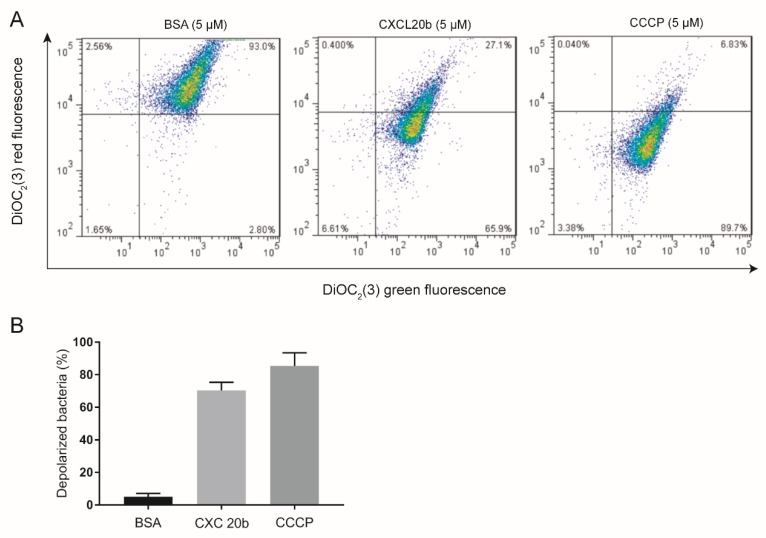
Grass carp CXCL20b destroys the integrity of bacterial cell membranes. (**A**) Flow-cytometry plots of 10,000 CFU *S. aureus* (ATCC 26923) incubated with 5 µM Bovine Serum Albumin (BSA; negative control), grass carp CXCL20b, and CCCP (positive control) using fluorescent dye DiOC_2_(3). (**B**) Normal and depolarized bacterial percentage of *S. aureus* (ATCC 26923) after treatment with 5 µM BSA, CXCL20b, and CCCP. Data from three independent experiments are shown (mean ± SEM).

To study the possible antimicrobial mechanism of CXCL20b, we used *S. aureus* (ATCC 25923) as a model bacterium. Given that the antibacterial mechanism of most AMPs and mammalian chemokines is based on their ability to disrupt bacterial membranes [30]. This leads to depolarization of the target bacteria membrane, which causes extracellular water to enter the cell, some electrolytes and molecules to flow out of the cell, and finally causes the death of the target bacterial cell. Hence we tested the effect of grass carp CXCL20b on the integrity of bacterial membrane. DiOC_2_(3) assay [31], which is commonly used to investigate cell membrane potential-dependent changes, was conducted to test the proportion of depolarized bacteria in a total of 10,000 CFU bacteria, which were treated with recombinant CXCL20b, carbonyl cyanide 3-chlorophenylhydrazone (CCCP positive control), and Bovine Serum Albumin (BSA; negative control; Figure 4A,B). The results showed that recombinant CXCL20b could obviously cause *S. aureus* cell membrane depolarization (65.9%), similar to CCCP (89.7%). By contrast, only a very low (2%) proportion of bacteria treated with BSA protein were depolarized. Thus, these data indicated that recombinant grass carp CXCL20b protein could obviously induce bacterial membrane depolarization.

### 2.5. Atomic Force Microscopy (AFM) Assay

**Figure 5:**
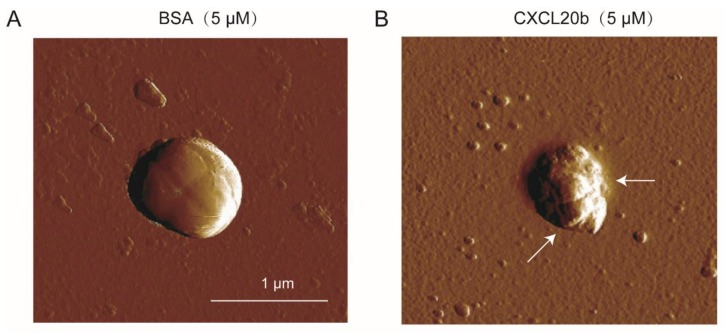
CXCL20b could cause *S. aureus* (ATCC 26923) cell membrane disruption in contrast to BSA (negative control). *S. aureus* (ATCC 26923) incubated with 5 µM BSA (**A**) and grass carp CXCL20b (**B**) for 15 min was observed by AFM. The arrow indicates the area of membrane rupture.

To further investigate whether grass carp CXCL20b protein can kill bacteria through membrane disruption, we used the atomic force microscopy (AFM) assay to observe the direct bacterial cell membrane disruption of recombinant CXCL20b protein-treated *S. aureus* (ATCC 25923). We incubated *S. aureus* (ATCC 25923) with 5 µM BSA protein and CXCL20b protein for 15 min. We washed off the protein and observed the bacterial morphology by AFM. In contrast to BSA-treated *S. aureus* (negative control; Figure 5A), we examined an obvious breakage of the cell membrane (arrows) on *S. aureus*, which was incubated with grass carp CXCL20b (Figure 5B). These results indicated that grass carp CXCL20b could kill bacteria through disruption of cell membranes, similar to most mammalian antimicrobial chemokines.

### 2.6. Structure Antimicrobial Assay of Grass Carp CXCL20b

**Figure 6:**
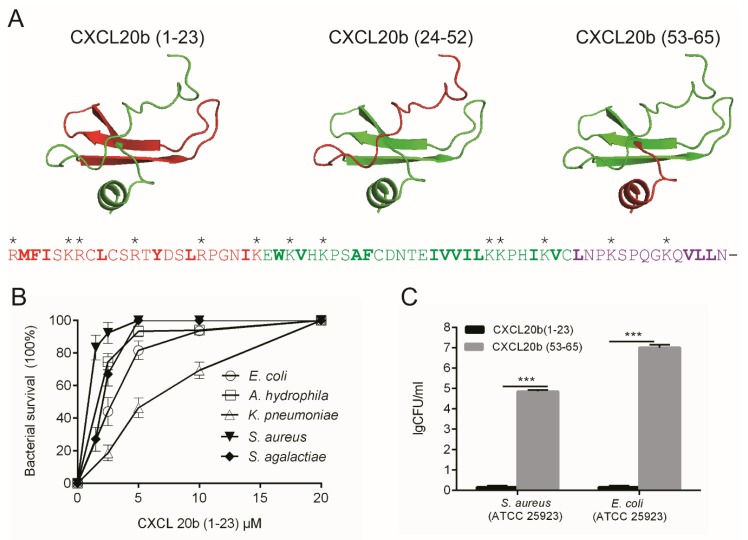
Sequence analysis and antimicrobial activity detection of grass carp CXCL20b fragments. (**A**) Secondary structure and sequence analyses. Target structure is in red, and other structures are in green. Sequences in red, green, and purple stand for the N-terminal random coil region (1–23), middle three antiparallel β-sheets region (24–52), and C-terminal α-helix region (53–65). Positive charge amino acids were marked by “*”. The hydrophobic amino acid residues were indicated in bold. (**B**) The antimicrobial activities of synthesized grass carp CXCL20b fragments were determined through CFU assays. N-terminal random coil peptide (1–23) with increasing concentrations was incubated for 30 min with Gram-positive bacteria *S. aureus* (ATCC 25923), *S. agalactiae* (ATCC 13813), and Gram-negative bacteria *E. coli* (ATCC 259220), *K. pneumoniae* (K13), and *A. hydrophila* (ATCC 7966). The middle β-sheets region (24–52) is insoluble. C-terminal α-helix region (53–65) did not show any antimicrobial activity against *S. aureus* (ATCC 25923) or *E. coli* (ATCC 25922) (**C**). Data from three independent experiments are shown as mean ± SD. *** *p* < 0.001.

Since certain classical AMPs [32] and chemokines share structural characteristics, such as C-terminal α-helix, disulphide bridges, and a high proportion of cationic amino acids [33], we asked whether the antimicrobial activity of grass carp CXCL20b is confined to one or more of these specific structural elements of the molecule. First, we analyzed the sequence and structure of grass carp CXCL20b. Our sequence and 3D model analysis indicated that this cytokine featured a typical mammalian chemokine structure, including N-terminal random coil (1–23), core structure containing three antiparallel β-strands (24–52), and C-terminal α-helix (53–65). In particular, the predicted grass carp CXCL20b had a relatively long N-terminal random coil and a relatively short C-terminal α-helix (Figure 6A). In addition, most cationic amino acids accumulated at the N-terminal and core structure containing three antiparallel β-strands (Figure 6A). We synthesized the three parts of grass carp CXCL20b. The synthesis of the core structure containing three antiparallel β-strand peptides CXCL20b (24–52) did not meet the requirement of insolubility in water. In addition, we found that the soluble CXCL20b protein could immediately change to insoluble flocculation when treated with DTT, reducing and alkylating the SH groups (data not shown). We speculated that the SH groups may play an important role in maintaining CXCL20b stability. Hence, we only tested the antimicrobial activities of CXCL20b (1–23) and CXCL20b (53–65) peptides. Through antimicrobial assay, we found that the N-terminal region peptides exerted potent antimicrobial activity against both Gram-positive bacteria and Gram-negative bacteria (Figure 6B). The tested MBC_90_ was approximately 1–8 μM. By contrast, we did not find any antimicrobial activity of the C-terminal α-helix region peptides even at a concentration of 65 μM (Figure 6C). Both CXCL20b and CXCL20b (1–23) peptides showed potent antibacterial activity against the above bacteria in 30 min. Thus, our data indicated that the antimicrobial activity of CXCL20b might be related to its N-terminal region.

## 3. Discussion

Several CXC-type chemokines, such as CXCL11 [23], CXCL8 [24], and CXCL12 [25] groups, exert chemotactic activity in bony fish. However, the CXCL20 group is part of the more recent chemokines whose function has not yet been investigated. The slow progress is partly due to low homology with mammalian chemokines. Here, we demonstrated an unexpected antimicrobial function of grass carp CXCL20b. This cytokine could efficiently kill both Gram-negative and Gram-positive bacteria within 30 min. AMPs exert antimicrobial activities primarily through mechanisms involving membrane disruption, so they have a lower likelihood of inducing drug resistance [34]. Extensive studies on the structure-activity relationship have revealed that net charge, hydrophobicity, and amphipathicity are the most important physicochemical and structural determinants endowing AMPs with antimicrobial potency and cell selectivity [35]. Here grass carp CXCL20b demonstrated the typical structure features of AMPs, with cationic and amphipathic properties (Figure 2), and microbicidal mechanism with membrane disruption (Figure 4 and Figure 5). We determined that grass carp CXCL20b was a novel fish AMP. It is noteworthy that the molecular weight of CXCL20b was about 7.5 KDa, bigger than most AMPs. As a potential antibacterial drug, the future research should focus on the safety and immunogenicity of the teleost-specific antimicrobial chemokines.

In mammals, many antimicrobial proteins have highly antimicrobial regions, such as the fifth helical region of human and mouse IFN-β [36]. In this article, we noted that the grass carp CXCL20b exhibited a long N-terminal random region, which contained large amounts of basic amino acid residues (Figure 6A). Using antimicrobial assay, we observed that synthesized N-terminal region peptides exerted potent antimicrobial activity against both Gram-negative and Gram-positive bacteria. On the contrary, the C-terminal region peptides of CXCL20b did not show any antimicrobial activity even at a high concentration of 65 μM (Figure 6C). It is unexpected, because the C-terminal helical region is core antimicrobial site of most antimicrobial chemokines [37]. For instance, the C-terminal helix region of three CXCL8 chemokines from murrel (*Channa striatus*), rainbow trout (*Oncorhynchus mykiss*) [38], and salmon (*Oncorhynchus*) [39] was revealed to exert direct antimicrobial activity. However, the N-terminal region of CXCL20b, not C-terminal helix is its core antimicrobial site. As many antimicrobial chemokines [33] or defensins [40] could exert potent antimicrobial activity after linearization. Hence it is possible that the antimicrobial effect of grass carp CXCL20b in vivo could be mediated independently by both N-terminal short linear regions and its tertiary structure.

Kinocidins are defined as chemokines and cytokines that have antimicrobial properties, many of which function as AMPs. In mammals, a few notable examples include the CXC-type chemokine family CXCL1, 4, 6, 7, 8, 9, 10, and 14; the CC-type chemokine family CCL1, 2, 5, and 8 [14]; and the cytokine IL-26 [31]. CK11 is a rainbow trout (*Oncorhynchus mykiss*) CC chemokine phylogenetically related to both mammalian CCL27 and CCL28 chemokines. More recently, this chemokine was reported to possess no chemotactic activity for unstimulated leukocyte populations but instead acts as a potent antimicrobial protein against bacterial pathogens [41]. Given our study, few teleost-specific groups CXCL20b may be another member of the kinocidin family, which acts only in a few bony fish.

In summary, intact recombinant grass carp CXCL20b was expressed and purified, which possessed cationic and amphiphilic features. CXCL20b shows the potent antimicrobial activity against both Gram-positive and Gram-negative bacteria by bacterial membrane disruption and depolarization mechanism. Furthermore, N-terminal random coil region, not C-terminal α-helix region, exhibited antimicrobial activity. The present study will strengthen the understanding of piscine chemokine in the immune system.

## 4. Materials and Methods

### 4.1. Expression and Purification of Recombinant Grass Carp CXCL20b Protein

Production of recombinant CXCL20b was carried out as described previously [42]. We used the bacterial expression system, which consisted of pET32a vector plasmid in *E. coli* strain BL21 trxB (DE3), to generate the recombinant Trx-CXCL20b protein. The transformed bacteria were cultured in Luria-Bertani broth medium overnight (with 100 µg/mL ampicillin and 25 µg/mL kanamycin). We then transferred the culture into fresh Luria-Bertani broth medium (1:100), which was incubated at 37 °C until OD_600_ reached 0.5–0.6. We added isopropyl β-D-1-thiogalactopyranoside into the culture at the final concentration of 1.0 mM to induce the expression of recombinant Trx-CXCL20b for 4–6 h. In particular, the low induced temperature (25 °C) could remarkably increase the expression of soluble protein Trx-CXCL20b. We collected the bacterial cells by centrifugation at 4000× *g* for 20 min. Bacteria were then resuspended in buffer A (20 mM Tris-HCl, 300 mM NaCl, and 50 mM imidazole, pH 7.8 at 25 °C). We used a high-pressure cracker to break the bacterial cells and release recombinant Trx-CXCL20b protein. Subsequently, we collected the supernatant by centrifugation at 40,000× *g* for 10 min. The recombinant Trx-CXCL20b proteins were purified by affinity chromatography (Ni NTA beads; Smart-Lifesciences). The recombinant Trx-CXCL20b proteins were eluted with buffer B (20 mM Tris-HCl, 300 mM NaCl, and 150 mM imidazole, pH 7.8 at 25 °C). The salt ions and imidazole were removed through gradient dialysis (3 kDa molecular mass cutoff Amicon ultracentrifugal filter device; Millipore, Billerico, MA, USA ). We used recombinant enterokinase (Smart-Lifesciences) to cleave off the redundant Trx-labeled proteins at 37 °C overnight. The recombinant CXCL20b was purified by cation exchange chromatography (SP Beads 6FF; Huiyan Biotechnology) in buffer C (20 mM Tris-HCl, pH 7.5 at 25 °C), extensively washed with buffer D (100 mM NaCl in 20 mM Tris-HCl, pH 7.5 at 25 °C), and eluted with 150–200 mM NaCl in 20 mM Tris-HCl (pH 7.5 at 25 °C). The grass carp CXCL20b was desalted in buffer E (20 mM Tris-HCl, pH 7.5 at 25 °C) by stepwise dialysis (3 kDa molecular mass cutoff Amicon ultracentrifugal filter device; Millipore) and then stored at −80 °C.

### 4.2. Microorganisms and Peptides

Gram-positive *S. aureus* (ATCC 25923), *S. agalactiae* (ATCC 13813), Gram-negative *E. coli* (ATCC 25922), and *A. hydrophila* (ATCC 7966) were obtained from American Type Culture Collection (ATCC). Gram-negative *K. pneumoniae* (K13) was isolated from clinical specimens identified by the Key Laboratory of Preventive Veterinary Medicine in Hubei Province (Wuhan, Hubei, China). All of the peptides derived from grass carp CXCL20b were chemically synthesized (Sangon Biotech, Shanghai, China).

### 4.3. Antimicrobial Assay

The antimicrobial activity of grass carp CXCL20b and CXCL20b (1–23) peptides were tested as described early [33]. Bacteria were cultured in Luria-Bertani broth medium (or brain heart infusion medium) to mid-logarithmic phase and washed twice with buffer A (10 mM Tris-HCl and 5 mM glucose, pH 7.4 at 25 °C). About 10^5^ CFU bacteria were incubated with buffer B (20 mM Tris-HCl, pH 7.5 at 25 °C) or different concentrations of recombinant grass carp CXCL20b protein or CXCL20b (1–23) peptides in a total of 100 µL. After 30 min (1 h for the MBC_90_ antimicrobial assay), the mixtures were poured onto Luria-Bertani solid medium and kept overnight. Percentage growth inhibition refers to the ratio of the colonies from CXCL20b treatment to the colonies from buffer B treatment. The number of colonies was counted by two independent investigators.

### 4.4. Fluorescence Microscopy

To study bacterial membrane potential loss, we used DiOC_2_(3) assay as previously described [31]. In brief, *S. aureus* (ATCC 25923) was cultured in brain heart infusion medium to mid-logarithmic phase. The bacteria were washed twice with buffer A (10 mM Tris-HCl, 10 mM NaCl, pH 7.4 at 25 °C) and then treated with 5 µM BSA (negative control), 5 µM CCCP (positive control), and 5 µM recombinant grass carp CXCL20b protein for 30 min. We used the DiOC_2_(3) dye (Invitrogen), a fluorescent membrane potential indicator, to stain *S. aureus* cells following the manufacturer’s protocol. Finally, *S. aureus* (10^5^ cells) per sample was prepared for fluorescence analysis.

### 4.5. Atomic Force Microscopy (AFM)

*S. aureus* (ATCC 25923) was cultured in the brain heart infusion medium to mid-logarithmic phase and then washed twice with buffer A (20 mM Tris-HCl, pH 7.5 at 25 °C). The washed bacteria were incubated with 5 µM recombinant grass carp CXCL20b or BSA for 15 min at 37 °C. Before imaging, we washed the bacteria twice with deionized water, transferred the cells to mica disks, and dried the disks overnight at 25 °C. AFM assay was carried out as previously described. AFM images were recorded on a Veeco Mulitmode AFM with NanoScope III controller operating in contact mode [43]. The data were analyzed with NanoScope Analysis software v.1.40 (Veeco, Santa Barbara, CA, USA). Scans were acquired at 25 °C at 1.0 Hz and 256 samples per line resolution.

### 4.6. Grass Carp CXCL 20b Modeling

Grass carp CXCL20b was modeled on SWISS-MODEL Template Library (https://www.swissmodel.expasy.org/). We downloaded the predicted protein structure model of grass carp CXCL20b as PDB files. We used PyMOL (PyMOL Molecular GrapHics System, Version 1.3, 2011; Schrodinger) to translate the PDB file into images. The net charge distribution on the grass carp CXCL20b and human CXCL1 surface was further determined with APBS software (https://www.easycounter.com/report/poissonboltzmann.org).

### 4.7. Statistical Analysis

The statistical analyses were performed using GraphPad PRISM 5.0 software. The comparisons between CXCL20b(1–23) and CXCL20b(53–65) were analyzed by an unpaired *t* test according to data distribution.

## 5. Conclusions

From the combination of the results, the grass carp CXCL20b was a cationic and amphipathic protein, which exerted potent antimicrobial activity *in vitro*. Similar with many natureAMPs, the CXCL20b protein could rapidly induce bacterial membrane depolarization and disruption. Additionally, the antimicrobial activity of CXCL20b was mainly relative to its N-terminal region. These results greatly enhanced our understanding of the function of few teleost-specific chemokine CXCL20b. The future steps to establish the studied compound for better use as an antimicrobial agent should include three aspects: (1). Evaluation of the cytotoxicity of CXCL20b protein to mammalian cells. (2). Investigation of whether CXCL20b have chemotaxis to mammalian leukocytes. (3). Evaluation of the immunogenicity of CXCL20b in the mouse model.

## Figures and Tables

**Table 1:** MBC_90_ determination of grass carp CXCL20b antimicrobial activities against several microorganisms in 1 h.

Bacteria Strain	MBC_90_ of CXCL20b (μM)	G^+^/G^−^
*Staphylococcus aureus* (ATCC 25923)	0.25	G^+^
*Streptococcus agalactiae* (ATCC 13813)	0.25	G^+^
*Escherichia coli* (ATCC 25922)	0.5	G^−^
*Aeromonas hydrophila* (ATCC 7966)	0.5	G^−^
*Klebsiella pneumoniae* (K13)	0.5	G^−^

## Supplementary Materials

Supplementary materials can be found at https://www.mdpi.com/2079-6382/9/2/78/s1. Figure S1. The structural features of human CXCL1.

## Abbreviations

DiOC_2_(3)	3,3′-Diethyloxacarbocyanine Iodide
AFM	Atomic Force Microscopy
BSA	Bovine Serum Albumin
MBC	Minimum Bactericidal concentration
PDB	Protein Data Bank
CCCP	Carbonyl cyanide 3-chlorophenylhydrazone
AMP	Antimicrobial peptide
NMR	Nuclear magnetic resonance
