# Supplementary material for: Characterization and Antimicrobial Activity of the Teleost Chemokine CXCL20b
Source: Antibiotics (Basel). 2020 Feb 12;9(2):78. doi: 10.3390/antibiotics9020078 (PMC7168194; doi:10.3390/antibiotics9020078)
Supplement: Supplementary file 1 [file antibiotics-09-00078-s001.pdf]

Supplementary Materials

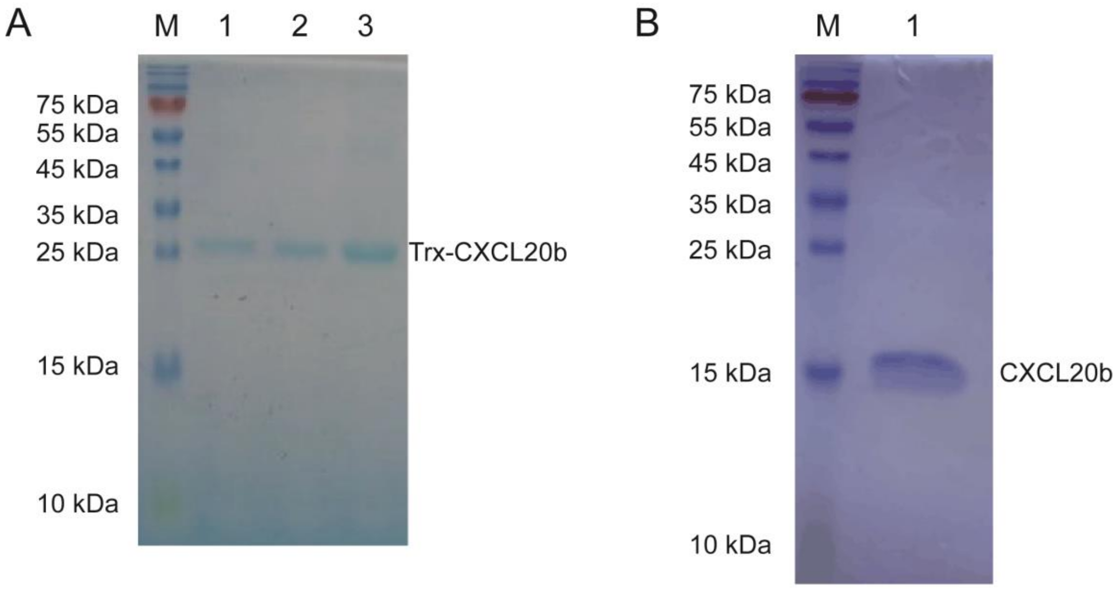

Figure S1. Purification of recombinant grass carp CXCL20b. (A) SDS-PAGE analysis of Trx-CXCL20b by the Ni-affinity chromatography purification. Lane M: protein molecular weight mark. Lane 1，2，3 show the purified Trx-CXCL20b protein from the Ni-NTA column. (B) SDS-PAGE analysis of CXCL20b protein by the Hitrap SP FF column purification. Lane M: protein molecular weight mark. Lane 1: purified CXCL20b protein from the Hitrap SP FF column.

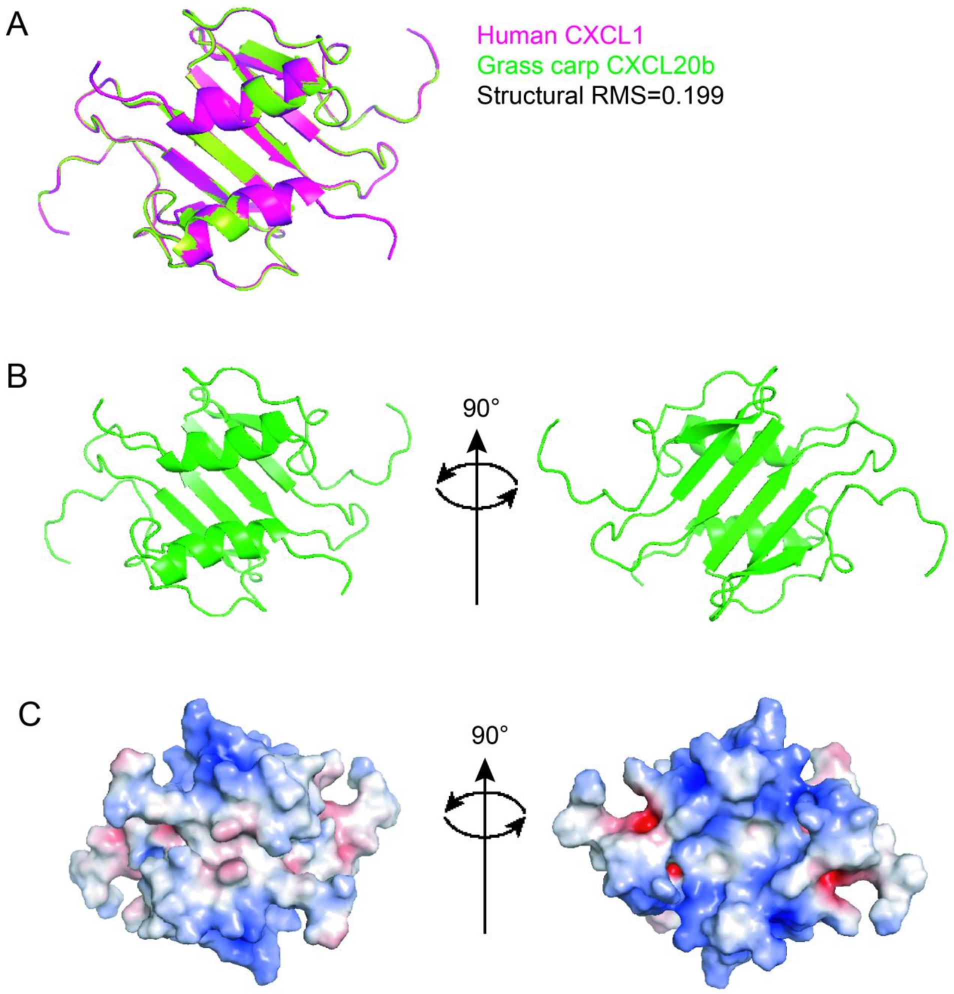

Figure S2 The structural comparison between human CXCL1 and grass carp CXCL20b. (A) Human CXCL1 and grass carp CXCL20b were compared at the structural level. Structural root-mean-square deviation (RMSD) is shown for overlay, which measures the average distance between atoms of superimposed human CXCL1 and grass carp CXCL20b. (B) On the basis of the NMR structure of human CXCL1, the images show a 3D model of human CXCL1 homodimers. (C) Electrostatic potentials mapped onto the surfaces of grass carp CXCL20b. Blue represents the cationic regions, red represents the negative regions, and white represents the hydrophobic residues.
